# Examination Questions for Red Cross Students

**Published:** 1914-02-14

**Authors:** 


### Examination Questions for Red Cross Students.

*To the Editor of* The Hospital.

Sir,—The letter from a lecturer to Red Cross students, published in your issue of January 31, raises a very important topic, and is very much to the point. While agreeing with her attitude, I should be glad to know whether she finds men or women examiners to be the greater sinners in going outside syllabuses and asking questions much too advanced for the pupils to answer. As one who has done several years of lecturing and examining for a county council in these subjects, I can say that there is a widespread belief among candidates, lecturers, and lecturers' assistants (who are usually trained nurses) that women examiners commit the vast proportion of these errors; that they are much more exigent and much less considerate of the candidates' circumstances, education, and status in general, and, in fact, a state of terror prevails whenever it is whispered that a class is to be examined by a woman and not by a man. I draw no inference from this at present; I merely ask whether the experience of others coincides with my own in this respect.—Yours faithfully,

Another Lecturer.

### The Altheat Stove.

*To the Editor of* The Hospital.

